# Understanding plant-microorganism interactions: The key roles of soil, rhizosphere, and direct and indirect mechanisms

**DOI:** 10.3934/microbiol.2025046

**Published:** 2025-12-19

**Authors:** Mohamed Hnini, Karim Rabeh, Malika Oubohssaine

**Affiliations:** 1 Microbiology and Molecular Biology Team, Center of Plant and Microbial Biotechnology, Biodiversity and Environment, Faculty of Sciences, Mohammed V University of Rabat, Avenue Ibn Battouta, BP 1014, Rabat 10000, Morocco; 2 Research Team in Science and Technology, High School of Technology Laayoune, Ibn Zohr University, Morocco; 3 Oasis System Research Unit, Regional Center of Agricultural Research of Errachidia, National Institute of Agricultural Research, PO. Box 415, Rabat 10090, Morocco

**Keywords:** Soil conservation, rhizosphere microbiota, plant-microorganism interactions, plant growth-promoting rhizobacteria (pgpr), sustainable agriculture

## Abstract

Soil, the Earth's upper crust layer, is crucial for ecological processes, comprising mineral, organic, and biological components that determine fertility and multifuncionality. Human-induced degradation necessitates advancements in pedology and soil conservation. The rhizosphere, surrounding plant roots, houses a diverse microbial community, notably bacteria, which enhance plant growth and disease resistance. Root exudates fuel biological activity and nutrient cycling, supporting microbial growth, improving soil structure, and reducing plant stress. Plant-microorganism interactions in ecological and agricultural systems play a vital role for maintaining primary production and ecosystem sustainability. Moreover, arbuscular mycorrhizae and nitrogen-fixing bacteria are essential, influencing plant development, sustainability, and ecosystem health. Specific bacterial phyla populate the rhizosphere and endosphere, with Plant Growth-Promoting Rhizobacteria (PGPR), such as *Pseudomonas* spp. and *Bacillus* spp., playing a prominent role. PGPR employ direct and indirect mechanisms, including phytohormone production, mineral solubilization, systemic resistance induction, antibiosis, competition for resources, and ACC deaminase activity, The amalgamation of these traits underscores the conceptual foundation for comprehending the ecological and agricultural implications of employing microbes. This inquiry is particularly relevant to sustainable agriculture, where the use of microbes, including PGPR, plays a crucial role in biofertilization and mitigating environmental stressors. Thus, investigating the ecological and agricultural implications through multi-omics approaches such as genomics, transcriptomics, proteomics, and metabolomics offers valuable insights. The integration of these multi-omics data provides a comprehensive framework for understanding the complex interactions between plants, bacteria, and fungi. This holistic perspective not only deepens our understanding of soil ecology but also lays the groundwork for informed and sustainable agricultural practices, fostering resilience against environmental stresses.

## 1. Introduction

Humanity is poised to confront a colossal challenge in providing sustenance to the burgeoning global population, projected to increase from 7.6 billion to 9.5–10 billion by the year 2050 [1]. Without expanding arable land surface or diminishing the use of pesticides and fertilizers, researchers must overcome this costly obstacle to avert further loss of natural ecosystems and depletion of natural phosphate deposits [2]. In most terrestrial ecosystems, nutrient scarcity has been thoroughly documented and poses a threat to plant growth and ecosystem stability [3]. Augmenting nutrient availability in the rhizosphere serves as a strategy employed by plants to combat this stress [4].

Boosting agricultural yields within agro-ecological systems is a complex endeavor, heavily influenced by diverse factors such as the agro-climatic environment, integrated approaches to management, and diverse cropping systems [5]. Depletion of water resources, salinity's impact on soils, water and environmental pollution, the global population increase, and the reduction of arable land pose threats to 21st-century agricultural sustainability [6]. The pursuit of enhanced agricultural productivity faces critical challenges beyond water scarcity, salinity, and environmental pollution. Nutrient shortages and the control of plant pests and diseases are key issues, impacting crop growth, yield, and nutritional content. Addressing nutrient deficiencies requires a comprehensive understanding of soil health and strategic interventions like precision agriculture. Additionally, effective pest and disease management is vital to prevent economic losses and threats to food security, necessitating sustainable strategies that balance biological, cultural, and chemical control methods [7]. To navigate the complexities of 21st-century agriculture, a holistic and integrated approach is essential. Collaborative efforts among researchers, policymakers, and practitioners are crucial to developing innovative solutions that address immediate concerns and nuanced issues, fostering resilience and sustainability in agro-ecological systems for a more secure global food system [8].

Effective water resource management strategies in arid and semi-arid regions must consider the impacts of climate change. Water conservation, irrigation water management, and nonconventional water use for agriculture are key issues to be considered in these areas [9]. Sustainable water management practices are essential to prevent irreversible damage to water resources and related resources such as soils and ecosystems [10].

It is well-acknowledged that this phenomenon could potentially exacerbate water resource crises in desert regions [11],[12]. Furthermore, it induces environmental stresses, notably drought and salinity, which are significant factors impeding plant growth and resulting in reduced agricultural productivity [13]. Drought is a slow-evolving disaster characterized by insufficient rainfall, leading to water deficits for plant growth. Drought can have severe consequences for agriculture and human health. It is estimated that droughts affect 55 million people worldwide each year, making it the greatest threat to livestock and crops globally [14].

Moreover, the rise in urbanization and detrimental human practices like deforestation, the over-application of pesticides and synthetic fertilizers, and the cultivation of multiple crops on farmlands contribute to environmental degradation. Similarly, industrial expansion, the overuse of fossil fuels, and deforestation have led to heightened emissions of greenhouse gases, thereby exacerbating global warming [15].

The vegetation in arid and semi-arid regions serves as a crucial renewable natural resource for the inhabitants, providing vital ecological services and sustenance. Thus, the inhabitants have relied on the vegetation to meet most of their nutritional needs [16]. In such environments, arid regions are particularly susceptible and prone to rapid degradation, leading to the deterioration of crucial soil attributes like soil structure, plant nutrient accessibility, organic matter concentration, microbial functions, and consequently, the disturbance of indigenous plant ecosystems [17]. In these challenging terrains, leguminous plants emerge as promising contenders for sustainable and environmentally sound initiatives to counterbalance the adverse impacts of soil degradation and climate change. Extensive research has demonstrated that Fabaceae intercropped with cereals effectively curbs soil erosion, minimizes the occurrence of soil-borne diseases, and enhances the nutrient composition of the soil [18]. Consequently, it is compelling to posit that leguminous plants represent one of the most encouraging elements of contemporary agricultural practices [19].

Apart from the morphological adaptations and physiological mechanisms employed by plants to withstand stressful conditions, soil microbial communities also play a pivotal role in fostering plant resilience to biotic and abiotic stresses. Notably, a group of beneficial soil-dwelling bacteria referred to as plant growth-promoting rhizobacteria (PGPR) significantly contribute to soil fertility and the promotion of plant growth through diverse biological mechanisms. These mechanisms include the enhancement of nutrient accessibility (such as N, P, K, and Mg), the stimulation of growth via phytohormone production, the reinforcement of stress tolerance (via activities like ACC deaminase and phytohormones), and the provision of heightened protection against phytopathogens through the production of siderophores, antibiotics, lytic enzymes, and the induction of systemic resistance [20].

Soil rhizosphere microbes, like those in other terrestrial systems, exhibit significantly different microbial communities from bulk soil, with a high abundance of taxa known to be endophytes [21], mycorrhizal fungi [22], and plant growth-promoting bacteria [23]. Moreover, plant roots are vital sources of carbon for microbial communities in an otherwise resource-limited and physically stressed matrix [24].

PGPR is a term coined by Kloepper in the 1970s [25] for the first time. This term refers to a variety of soil bacteria groups, including *Alcaligenes, Arthrobacter, Azospirillum, Bacillus, Bradyrhizobium, Burkholderia, Azotobacter, Klebsiella, Mesorhizobium, Enterobacter, Flavobacterium, Pseudomonas, Rhodococcus, Serratia, Streptomyces*, and *Variovorax*. These bacteria are key components of soil-plant systems, engaging in a complex network of interactions in the rhizosphere, thereby affecting plant growth and yield.

Our understanding of plant-associated microbe functions has been constrained by the limited diversity represented in cultured microbial communities. Only 1% of soil bacteria are culturable under specific conditions [26], and cultured microbes may behave differently in natural environments compared to laboratory settings [27]. The presence of microbial species in field soils introduces competition, impacting the behavior of microbes on host plants. Technical advances in molecular biology, such as gene sequencing technologies like 16S rRNA amplicon sequencing and shotgun metagenome sequencing, address these gaps by determining the abundance of microbial species in environmental samples [28].

The intricate relationship between plant hosts, soil, and microbiota is crucial for maintaining healthy ecosystems and sustainable agriculture. Omics technologies enable a molecular-level analysis of these interactions, offering insights to enhance plant health and improve soil ecosystem functions. Traditional ecological health studies often rely on time-consuming and biased laboratory culturing methods that may not capture the full extent of microbial diversity or identify functional potential [29].

In this review, we focus on the potential of leguminous plants, rhizobial and arbuscular mycorrhizal symbiosis, and PGPR in addressing challenges like soil degradation, climate change, and resource scarcity, particularly in arid regions. It emphasizes the role of root microbiota in sustaining microbial communities and explores the use of advanced omics approaches to better understand plant disease ecology and microbiome dynamics. By integrating these perspectives, we aim to enhance knowledge in soil microbiology, plant-microorganism interactions, and sustainable agriculture, laying the groundwork for future research and applications.

## 2. Soil and the Rhizosphere

### 2.1. Soil

The soil constitutes the upper part of the Earth's crust, comprising mineral, organic, and biological fractions that interact with one another to determine soil richness and fertility. As a fundamental component of the ecosystem, soil plays a pivotal role as a controller and revealer of numerous short- and long-term ecological processes through its physical, chemical, and biological properties [30]. Furthermore, soil is indispensable for the proper functioning of terrestrial ecosystems and represents a valuable resource that must be protected due to its accelerated degradation, often attributed to human activities. The safeguarding of this vital resource primarily necessitates the development of research in the crucial field of pedology [31].

### 2.2. The Rhizosphere

The rhizosphere, a term coined by Lorenz Hiltner [32], refers to the region surrounding plant roots. “Rhizo” derives from the Greek “rhiza,” meaning “root,” and “sphere” denotes the field of action or influence [33]. This area holds vital importance for plant survival and growth as it constitutes a specific environment where material and energy exchanges between soil and the plant are particularly active [34].

The rhizosphere houses a microbial density exceeding one million microorganisms per gram of soil, characterized by intense microbial activity, and is largely influenced by the roots of various plant species [35]. Among the bacteria present in the rhizosphere, some contribute to plant growth through various mechanisms. The beneficial effect of these microbial species has been known for several decades, and they are commonly used to enhance plant growth and protect them against diseases [36].

The rhizosphere can be divided into three distinct parts:

- The rhizosphere, corresponding to the soil surrounding the plant roots (rhizosphere proper). In this zone, exchanges occur between the soil, roots, microorganisms, and associated fauna [37]. These exchanges manifest as bidirectional flows of water and nutrients.
- The rhizoplane, which is the interface between the root and the soil.
- The endorhizosphere, representing the internal zone of the root.

The release of substrates by plant roots characterizes the rhizosphere and influences microbial activity in the surrounding soil, a phenomenon known as the rhizospheric effect. This effect fosters the coexistence of numerous microorganisms, including bacteria, protozoa, and algae, with bacteria being particularly abundant and exerting a significant influence on plant physiology.

Plants exploit the water and nutrient resources necessary for their development and growth in the rhizosphere. Up to 30% of the compounds photosynthesized by the plant are reintroduced into this zone through a process known as root exudation. Root exudates contain a diversity of complex organic compounds, such as organic acids, sugars, growth regulators, isoflavonoids, and enzymes. These organic nutrients are utilized by microorganisms present in the rhizosphere, either for their own growth or degraded into CO_2_ [38]. Various substances secreted by roots stimulate microbial activity, including carbohydrates (sugars and oligosaccharides), organic acids, vitamins, amino acids, flavonoids, enzymes, and volatile compounds [33], peptides, proteins, and phytohormones [39]. The rhizosphere is thus characterized by remarkably high biological activity [40]. Furthermore, root exudates have several beneficial effects, such as increasing soil aggregation, limiting mineral leaching [41], detoxifying heavy metals (Jones and Hinsinger, 2008), and reducing root tip desiccation by limiting friction between the soil and the root during growth [42].

**Figure 1.**
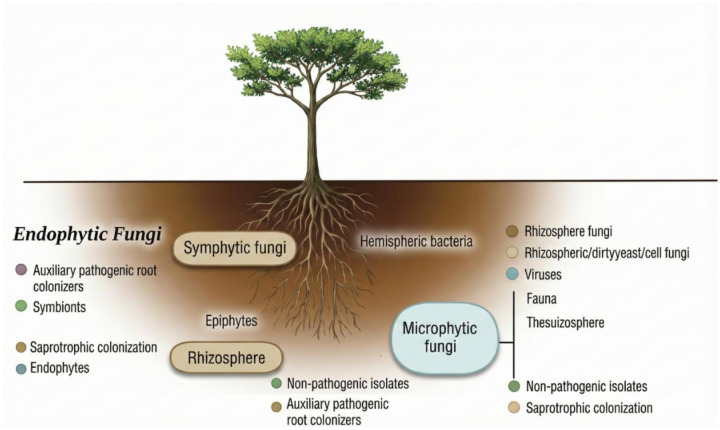
Symbiotic and free-living microbes in the rhizosphere.

The rhizosphere harbors a wide variety of microorganisms, including archea, earthworms, nematodes, protozoa, fungi, algae, and bacteria (Figure 1) [40]. Among them, bacteria are the most abundant and are likely to have a significant influence on plant physiology. The composition of the rhizosphere varies depending on the specific characteristics of each plant, the density of its root system, surface properties, as well as the physicochemical properties of the soil [43].

### 2.3. Rhizospheric rhythms: Unveiling the impact of plant-bacteria-fungal interactions on soil dynamics

Plant–bacteria–fungi interactions are inherently complex and dynamic, playing essential roles in ecosystem functioning, plant health, and nutrient cycling. These interactions encompass direct physical associations and indirect signaling mechanisms. In agroecosystems, the soil microbiome is a critical determinant of soil fertility, crop productivity, and plant resilience to biotic and abiotic stressors [44]–[46]. These microbial communities are closely linked to soil structure particularly aggregation and pore connectivity which governs the movement of water, oxygen, and nutrients [47]. The rhizosphere represents a highly favorable niche for microbial colonization [48]. However, the distribution of these microbes in the soil is far from random. The soil, acting as a diverse substrate, operates as a filter, selectively hindering the settlement of specific microorganisms. Consequently, environmental filters, such as soil physicochemistry or nutrient content, exert influence over the ability of microorganisms to establish themselves in the rhizosphere [49]–[51]. Studies by Edwards et al., (2023) have demonstrated that soil texture defined by its clay, silt, and sand composition significantly affects microbial biomass and activity [52]. Notably, soil type was found to have a greater impact on microbial community structure and rhizodeposition utilization than seasonal variation or management practices. Moreover, accumulating evidence indicates that soil pH is a key environmental filter shaping microbial communities [53],[54].

Numerous studies have demonstrated that soil conditions significantly influence the structure of the soil microbiome. However, less attention has been given to the effects that microorganisms have on their soil environment, with much of the research focusing on microbial roles in carbon and nitrogen cycles. Beyond their involvement in nutrient cycling and organic matter transformation, soil microbes actively modify their habitat through a range of biochemical and biophysical processes. These microbially induced alterations in soil properties can locally shape microbiome composition, carrying important ecological consequences [55],[56]. Liu et al., (2023) show that biotic filters, such as plant affinity and interactions between microbes, also play a role in the establishment of a microbial community [57].

Certain microbial taxa exhibit greater competitive abilities, enhancing their likelihood of successful establishment in the rhizosphere. This advantage may stem from superior nutrient acquisition strategies or a heightened capacity to overcome environmental filtering mechanisms [58]. Once established, the degree of interaction between microorganisms and the host plant can vary significantly, influencing microbial function and community dynamics [59],[60]. The translocation of photosynthetically derived carbon from the plant to the soil creates a spatial and functional gradient of rhizodeposition. This spatial heterogeneity in resource availability drives niche differentiation and microbial specialization along the root-soil interface [61].

## 3. Plant-Microorganism Interactions

### 3.1. Generalities

Living organisms constantly interact with each other within their environment. Such interactions are instrumental in shaping the growth and persistence of the organisms concerned. Interactions between plants and the microorganisms associated with their root systems can range from pathogenic to neutral or beneficial. Particularly in the realms of ecology and agriculture, the most advantageous interactions for plants occur with arbuscular mycorrhizae and root nodules [62]. Notably, filamentous actinobacteria are also regarded as one of the key constituents within the rhizospheric microbiota [63].

### 3.2. Symbiotic interactions

#### 3.2.1. Biological nitrogen fixation

Nitrogen is an essential element for plant growth and development, as it is a component of proteins, urea, nucleic acids, polyamines, and acts as an enzymatic cofactor, among other functions. These molecules are present in all living cells [64]. Nitrogen assumes a critical role in photosynthesis as the primary component of chlorophyll, which allows plants to capture the necessary light energy for their growth and biomass production. Nitrogen, in the form of gas, constitutes approximately 78% of the atmospheric air. However, only forms such as nitrate (NO₃⁻) and ammonium (NH₄⁺) can be utilized by plants, and both are present in limited quantities in the soil. Therefore, nitrogen can become a limiting factor for plant production, much like water [65].

Nitrogen-fixing bacteria can convert atmospheric nitrogen (N₂) into ammonia using a specific enzyme called nitrogenase. Three categories of nitrogen-fixing bacteria are distinguished: (1) Cyanobacteria, responsible for 40 to 50% of biological nitrogen fixation, (2) soil bacteria free-living or (3) bacterial symbiotic with plants. However, few species are capable of forming a nitrogen-fixing symbiosis, which led to the development of industrial nitrogen fertilizer production by Fritz Haber and Carl Bosch in the early 20th century [66]. While nitrogen fertilizers are extensively employed in agriculture, their use has been associated with adverse effects on the environment and human health. Consequently, it is imperative to explore alternative approaches that can curtail the dependence on nitrogen fertilizers without compromising high production yields. One such strategy involves the cultivation of legumes, which possess the unique capability to efficiently fix and mobilize nitrogen, thus lessening the necessity for nitrogen fertilizers [67].

#### 3.2.2. Rhizobial symbiosis

Symbiosis refers to a situation in which two or more organisms from different species live in close interaction for an extended period [68]. The symbiotic relationship between Rhizobium and legumes represents one of the most extensively examined plant-microorganism interactions, owing to its pivotal role in nitrogen fixation within nearly all agricultural systems [69].

##### 3.2.2.1. Legumes

Legumes (family Fabaceae, order Fabales) are a diverse group of dicotyledonous plants that play critical ecological and economic roles. Comprising approximately 765 genera and 19,500 species, Fabaceae is one of the largest and most widely distributed angiosperm families (LPWG, 2017; Legume Phylogeny Working Group, [70]). It is the most prevalent plant family in both humid tropical and dry forests of the Americas and Africa [71].

The family exhibits remarkable morphological and ecological diversity, ranging from herbaceous annuals and shrubs to tropical trees and woody climbers. Legumes are utilized globally for food, fodder, timber, oil, and medicine. In many developing countries, they are a primary source of dietary protein [72] and contribute carbohydrates, fiber, vitamins, minerals, and essential fatty acids [73]. Legumes are adapted to a wide range of environments, from Arctic herbs to xerophytic shrubs and equatorial forest trees, underscoring their global ecological breadth [74],[75].

Traditionally classified into three subfamilies based on floral morphology [76]. Fabaceae is now divided into six subfamilies, *Caesalpinioideae, Cercidoideae, Detarioideae, Dialioideae, Duparquetioideae*, and *Papilionoideae*, based on comprehensive chloroplast and nuclear genomic analyses [77],[78].

Ecologically, legumes are vital for their ability to fix atmospheric nitrogen via symbiosis with Rhizobium spp. in root nodules. This symbiotic process converts inert N₂ into bioavailable nitrogen compounds, enhancing soil fertility and benefiting surrounding plant communities after root decomposition [79].

##### 3.2.2.2. Rhizobia

The term “*rhizobium*” is used to refer to bacteria capable of inducing nodule formation on the roots or stems of host leguminous plants and fixing atmospheric nitrogen (N₂) for their host plants in exchange for carbon [80]. Rhizobia are Gram-negative, rod-shaped bacteria, measuring approximately 0.5 to 0.9 µm in width and 1.2 to 3 µm in length. Fast-growing rhizobia have 2 to 6 mobile peritrichous flagella, while slow-growing rhizobia have a single polar or subpolar flagellum [81].

Rhizobia belong to the Alpha and Beta clades of the *Proteobacteria* class. Currently, there are 18 genera of rhizobia, containing over 250 validly named and described species, and this number continues to increase rapidly [82]. The main genera of nitrogen-fixing bacteria associated with the root nodules of legumes belong to α-Proteobacteria in the families Rhizobiaceae (*Rhizobium, Ensifer, Allorhizobium, Pararhizobium, Neorhizobium, Shinella*), Phyllobacteriaceae (*Mesorhizobium, Aminobacter, Phyllobacterium*), *Brucellaceae* (*Ochrobactrum*), Methylobacteriaceae (*Methylobacterium, Microvirga*), Bradyrhizobiaceae (*Bradyrhizobium*), Xanthobacteraceae (*Azorhizobium*), and Hyphomicrobiaceae (*Devosia*). Some also belong to the β-Proteobacteria in the Burkholderiaceae family (*Paraburkholderia*, *Cupriavidus*, *Trinickia*) [83],[84]. It is worth noting that rhizobial bacteria are not the only inhabitants of legume root nodules; they coexist with non-symbiotic endophytic bacteria [85].

##### 3.2.2.3. Establishment of the rhizobium-legume symbiosis

The establishment of the symbiotic association between rhizobia and legumes involves a sophisticated exchange of signals between the bacterium and the plant. Notably, legumes possess the capacity to form a symbiotic relationship with soil-dwelling bacteria called rhizobia, which have the remarkable ability to fix atmospheric nitrogen [86]. This symbiosis prompts the development of nodules on the plant's roots, creating a conducive environment for the conversion of atmospheric nitrogen into ammonia by the bacteria, a form of nitrogen readily assimilable by the plant [87].

The successful establishment of this symbiotic interaction between rhizobia and legumes is contingent upon genetic and molecular mechanisms that confer symbiotic specificity. This specificity operates at the species and genotype levels, indicating that no single strain of rhizobium can establish a symbiotic relationship with all legumes, and vice versa. The underlying mechanisms of legume-rhizobium symbioses continue to be the subject of extensive research [69],[87].

#### 3.2.3. Structural biology advances in rhizobial symbiosis signaling

The establishment of symbiosis is predicated on a complex molecular dialogue that ensures precise host-symbiont compatibility. This dialogue begins with plant flavonoids activating rhizobial nod genes (notably via NodD regulators), leading to the synthesis of specific lipo-chitooligosaccharides known as Nod factors. These factors are recognized by specific LysM receptor kinases on the plant root epidermis, a critical first step that triggers root hair deformation, infection thread formation, and cortical cell division, leading to nodule organogenesis [88]. The structural specificity of Nod factors dictates which legume species can be successfully nodulated, with some rhizobia possessing multiple nodD genes to broaden host range or enhance competitiveness [89].

While these general principles of symbiotic specificity are established, the last two years have witnessed significant breakthroughs in elucidating the structural architecture and molecular regulation of the signaling interface. High-resolution structural biology and advanced genetic analyses have refined our understanding of how legumes perceive lipo-chitooligosaccharides (LCOs) and initiate the signaling cascade.

##### 3.2.3.1. Receptor complex architecture and specificity

The perception of Nod factors relies fundamentally on Lysin Motif Receptor-Like Kinases (LysM-RLKs). Phylogenetic and structural studies have expanded the known repertoire of these receptors. Specifically, the LYR-IB clade has been identified as a high-affinity receptor group for both LCOs and chitooligosaccharides, functioning complementarily to the previously characterized LYR-IA group to broaden the specificity of perception across angiosperms [90],[91].

Furthermore, nanobody-driven approaches have definitively resolved the core receptor complex architecture in *Lotus japonicus*. It is now confirmed that the Nod factor receptors NFR1 and NFR5 form a heteromeric complex where kinase activity is obligate for the initiation of nodule organogenesis and infection threads [92].This mirrors the LYK3-NFP complex functionality in *Medicago truncatula*, suggesting a conserved structural paradigm across legumes [93]. Advancements in computational biology, specifically molecular dynamics and machine learning, are now facilitating the prediction of these receptor-ligand affinities with unprecedented accuracy, mapping substrate specificity even for flexible LCO molecules [94].

##### 3.2.3.2. Spatiotemporal regulation and host-microbe interplay

The assembly and activity of these receptor complexes are subject to rigorous spatiotemporal regulation. The recruitment of Nod factor receptors to the root hair tip a critical step for infection thread formation is orchestrated by scaffold proteins such as RinRK1 and Flotillin 1, which facilitate receptor localization into nanodomains [95]. Conversely, regulatory proteins like the Bax inhibitor GmBI-1\alpha interact with the receptor kinase NFR1\alpha to modulate nodule number and infection efficiency, preventing hyper-nodulation [96].

The regulation of this pathway also involves a dynamic “arms race” between host and microsymbiont. Recent evidence demonstrates that the rhizobial effector NopT can directly cleave the NFR5 receptor to dampen signaling. In a display of co-evolutionary adaptation, the host kinase NFR1 can phosphorylate and inactivate NopT, thereby restoring signaling integrity [97]. Furthermore, signal modulation is achieved through the endocytosis of the co-receptor SYMRK; this rhizobia-induced internalization is essential for controlling the amplitude and duration of the symbiotic signal [98].

##### 3.2.3.3. Downstream transduction and biotic interference

Following receptor activation, the signal is transduced to the nucleus via a cascade involving calcium oscillations. Early Phosphorylated Protein 1 (EPP1) has been identified as a critical link in this chain, required for calcium spiking and the activation of the early infection program [99]. However, this pathway is vulnerable to biotic interference; structural studies have revealed that pathogens, such as soybean cyst nematodes, secrete LCO-hydrolyzing enzymes (e.g., HgCht2) to enzymatically degrade signaling molecules and disrupt the establishment of symbiosis [100].

#### 3.2.4. Arbuscular mycorrhizal symbiosis

Given the heterogeneous spatial distribution of nutrients in soils [101], the proliferation of root and mycorrhizal hyphae in nutrient-rich regions represents a prevalent adaptive strategy to facilitate the swift acquisition of nutrients [102]. This mechanism not only fosters plant growth but also imparts a competitive edge to species exhibiting heightened root plasticity [103].

Arbuscular mycorrhizal fungi (AMF) are widely distributed soil microorganisms that establish a mutually beneficial symbiotic relationship with most terrestrial plants, accounting for over 80% of plant species in terrestrial ecosystems [104]. These fungi enhance the nutritional status of plants by bolstering the uptake of vital nutrients like nitrogen and phosphorus [105]. They are also known to facilitate the absorption of low-mobility mineral nutrients from the soil, particularly phosphate, thereby improving overall plant nutrition and growth [106]. The establishment of this symbiosis not only augments plant resilience to abiotic and biotic stresses, including metal stress [107] but also enhances plant tolerance to heavy metals through various mechanisms, such as ameliorated nutrient supply or reduced water stress [108].

To explore the potential of AMF in agriculture, some challenges and prospects need to be considered. The specificity of the legume-rhizobium symbiosis occurs at species and genotypic levels, with certain bacterial strains being able to infect and nodulate one host plant but not another [109]. Therefore, it is essential to identify the most suitable AMF strains for specific crops and environmental conditions. Additionally, the long-term effects of agricultural management practices on AMF need to be considered, as they can impact the composition and diversity of AMF communities in crop fields [110].

### 3.3. PGPR

The microbial community inhabiting the rhizosphere differs significantly from microbial communities in both root tissues of the plant (endosphere) and non-rhizospheric soil [111], the rhizosphere denoting the soil region immediately surrounding the roots [112], typically hosts a greater abundance of bacteria. Moreover, the root microbial community predominantly comprises species from the phyla *Pseudomonadota, Actinomycetota, Bacteroidota*, and *Bacillota*, previously recognized as *Proteobacteria, Actinobacteria, Bacteroidetes*, and *Firmicutes* [113],[114], respectively, recently renamed by Oren and Garrity (2021) [115].

The term “PGPR” encompasses different groups of soil bacteria such as *Alcaligenes, Arthrobacter, Azospirillum, Bacillus, Bradyrhizobium, Burkholderia, Azotobacter, Klebsiella, Mesorhizobium, Enterobacter, Flavobacterium, Pseudomonas, Rhodococcus, Serratia, Streptomyces*, and *Variovorax*. These bacteria play a pivotal role in soil-plant systems, engaging in intensive interactions within the rhizosphere and significantly influencing plant growth and yield.

**Figure 2.**
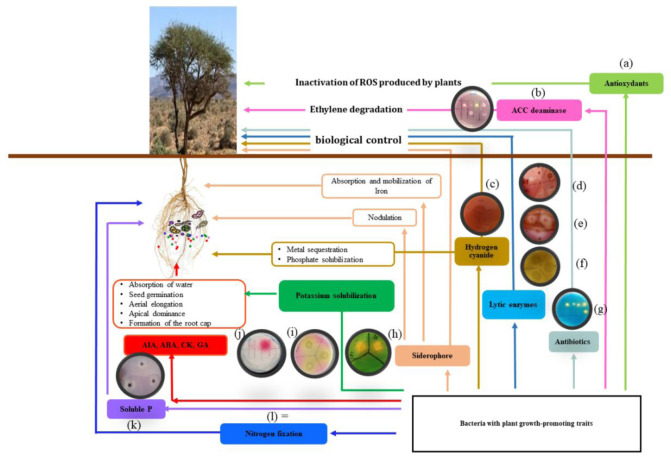
Characteristics of the major Plant Growth-Promoting Microbes (PGP) involved in improving stress tolerance and crop productivity in various agroecological conditions (rectangular boxes represent various aspects of pgp activities) while the circle represents some examples of those pgp traits on solid media: (a) Antioxydants [118], (b) ACC deaminase Palaniyandi et al. (2014)., (c) Hydrogen cyanide [120], (d) Chitinase [121], (e) Cellulase [122], (f) Protease [123], (g) Antibiotics [124], (h) Siderophores [125], (i) Potassium [126], (j) Auxin [127], (k) Phosphate (Pikovskaya and Pikovskaya 1948), and (l) Nitrogen fixation [23].

Rhizobacteria, as heterotrophic organisms, depend on organic compounds as an energy source. In the rhizosphere, these bacteria find ample satisfaction as they use various substrates from the host plant, such as detached root cortical and epidermal cells, root mucilage polysaccharides, sugars, and organic amino acids from root exudates, among others [112]. The abundant proliferation of bacteria in the soil is due to their ability to multiply rapidly and utilize a wide variety of substrates as sources of energy and nutrients [116]. PGPR, representing a very small fraction of rhizobacteria (2-5%), have a beneficial effect on plant growth (Glick, 2012). Figure 2 illustrates the mechanisms by which rhizospheric bacteria can increase yield, inhibit pathogens, reduce the presence of contaminants, tolerate stress, and produce beneficial substances and molecules [117].

PGPR microbes are a heterogeneous group of bacteria known for producing and secreting regulatory chemicals near plant roots, which promote plant growth [129]. Some PGP microbes can synthesize phytohormones that influence root system development or molecular signals that interact with plant roots [130]. Additionally, some of these bacteria contribute to the overall health of plants by improving the acquisition of nutrients by host plants, providing protection against phytopathogens, and fostering resistance to various abiotic stresses [131] (Figure 2).

Different strains of PGPR microbes are capable of increasing crop yields, providing biological control, enhancing resistance to foliar pathogens, promoting nodulation in legumes, and improving seedling emergence [132]. Most bacterial strains involved in these beneficial activities are Gram-negative bacteria, and they belong to various bacterial taxonomic groups. Some of the most frequently isolated and identified genera *Acinetobacter, Aeromonas, Agrobacterium, Alcaligens, Allorhizobium, Arthrobacter, Azoarcus, Azorhizobium, Azospirillum, Azotobacter, Bacillus, Beijerinckia, Bradyrhizobium, Burkholderia, Caulobacter, Chromobacterium, Delftia, Enterobacter, Erwinia, Flavobacterium, Frankia, Gluconacetobacter, Klebsiella, Mesorhizobium, Micrococcus, Paenibacillus, Pantoea, Pseudomonas, Rhizobium, Serratia, Streptomyces*, and *Thiobacillus* [133],[134].

#### 3.3.1. Genotype-dependent responses to PGPR

The assembly of the rhizosphere microbiome is not a stochastic process; rather, it is strictly governed by host genetic traits. Plant genotype dictates the recruitment and maintenance of PGPR through three primary mechanisms: the modulation of root exudate chemistry, the structural complexity of the root system architecture (RSA), and the variability of innate immune signaling pathways [135].

##### 3.3.1.1. Chemotactic selection via root exudate profiles

The metabolic distinctness of a plant genotype acts as a primary selective force in the rhizosphere. Plants secrete a diverse array of carbon-rich compounds, including sugars, organic acids, and secondary metabolites, which serve as chemotactic signals and nutrient sources for specific microbial taxa [136].

Investigations demonstrate that intraspecific genetic variation significantly alters these exudate profiles. For instance, Pacheco-Moreno et al., (2024) observed that modern barley (*Hordeum vulgare*) cultivars exhibit distinct exudation patterns compared to older genotypes, directly correlating with the differential recruitment of beneficial *Pseudomonas* species [137]. Similarly, Anderson et al., (2024) highlighted that novel carrot genotypes possess unique trait diversities that influence soil microbial assembly through specific exudate compositions [137].

Furthermore, specific metabolites function as precise distinct filtering agents. For instance, in maize, root-derived flavones have been shown to selectively enrich members of the Oxalobacteraceae, enhancing nitrogen acquisition capabilities [138]. Additionally, the concentration of signaling compounds such as jasmonic acid and sugars in exudates varies significantly across developmental stages and genotypes, acting as determinants for the rhizobacterial community structure [139].

##### 3.3.1.2. Physical niches and root system architecture

Beyond chemical signaling, the physical configuration of the root system plays a crucial role in microbial colonization. Genetic variation in root system architecture (RSA) encompassing traits such as root length density, branching angles, and lateral root proliferation creates heterogeneous physical microenvironments [140].

These morphometric traits define the spatial distribution of resources and potential attachment sites for PGPR. Galindo-Castañeda et al., (2022) argue that capitalizing on synergies between specific root phenotypes and root-associated microorganisms is essential for optimizing soil resource uptake [141]. Moreover, PGPR can reciprocally influence these architectural traits; for example, *Pseudomonas* sp. CM11 has been found to specifically induce lateral root development through the modulation of PLETHORA-dependent pathways, suggesting a bidirectional feedback loop between host genotype and microbial partners [142].

##### 3.3.1.3. Immunological compatibility and signaling

The compatibility between a host plant and beneficial microbes is also gated by the plant's innate immune system. Genetic loci governing the production and perception of defense hormones specifically salicylic acid (SA), jasmonic acid (JA), and ethylene vary by genotype and influence the plant's “responsiveness” to PGPR [143].

Effective colonization often requires PGPR to evade or suppress local immune responses while triggering Induced Systemic Resistance (ISR). Wu et al., (2018) demonstrated that *Bacillus amyloliquefaciens* produces elicitors that interact synergistically with plant signaling pathways to bolster systemic immunity [144]. However, this interaction is genotype-dependent; natural genetic variation in Arabidopsis has been linked to differential growth promotion and ISR capacity, suggesting that breeding programs must account for these immunological “compatibility traits” to fully exploit microbial benefits [145].

#### 3.3.2. Direct mechanisms of PGPR action

##### 3.3.2.1. Phytohormone production

Phytohormones are signaling molecules that regulate plant growth, development, and stress responses, even at very low concentrations [146]. Among the most important are auxins, cytokinins, and gibberellins, which modulate key processes such as cell division, apical dominance, and organogenesis [147].

Numerous rhizobacteria produce phytohormones, notably indole-3-acetic acid (IAA), a key auxin. Beneficial genera such as *Rhizobium*, *Bradyrhizobium*, *Azospirillum*, and *Bacillus* typically synthesize IAA via the indole-3-pyruvic acid (IPyA) pathway [148]. In contrast, some phytopathogens use the indole-3-acetamide (IAM) pathway [149]. IAA-producing PGPR enhance root elongation, nutrient uptake, and overall plant biomass by influencing hormone signaling and activating IAA-responsive genes [150].

##### 3.3.2.2. Siderophore-mediated iron acquisition

Iron is essential for plants and microorganisms due to its role in redox reactions and enzyme functions, yet its bioavailability is limited under aerobic and neutral pH conditions due to its tendency to form insoluble compounds [151]. To access iron, many soil microorganisms, including PGPR, secrete siderophores low molecular weight chelating compounds with high affinity for ferric iron (Fe³⁺) [152].

Siderophores scavenge iron from mineral and organic complexes, enabling bacterial uptake through specific transport systems [153]. Their synthesis is tightly regulated by iron availability, and they are classified based on their iron-chelating functional groups, including hydroxamates, catechols, and α-hydroxyl-carboxylates [154]. In the rhizosphere, PGPR-produced siderophores not only enhance bacterial survival but also improve iron uptake by plants, thereby contributing to plant health and productivity.

##### 3.3.2.3. Solubilization of mineral elements

###### 3.3.2.3.1. Phosphate solubilization

Phosphorus (P) is a ubiquitous element in the natural environment. Alongside nitrogen (N) and potassium (K), it is regarded as a fundamental constituent of both plant and animal life. Phosphorus assumes a crucial role in plant metabolism and stands as one of the indispensable nutrients necessary for the growth and development of plants [155]. Plants primarily absorb phosphorus during their vegetative growth phase, with a significant portion being transported to fruits and seeds during the reproductive stages.

In soil, the fraction of available phosphorus primarily exists as monovalent dihydrogen phosphate (H₂PO₄⁻) and divalent hydrogen phosphate (HPO₄²⁻) anions, which predominate at near-neutral pH (6–7), each contributing roughly equally under such conditions [156]. However, the vast majority of total soil phosphorus (approximately 95–99%) is bound in insoluble mineral complexes, such as iron, aluminum, and calcium phosphates, and is therefore unavailable for plant uptake [157].

In practical agriculture, given that phosphorus content in soils varies from 0.4 to 1.2 g per kg of soil [158], it is necessary to use fertilizers based on soluble phosphates to achieve optimal crop yields. However, since these fertilizers can easily precipitate as insoluble complexes, repeated and excessive application is often required in cultivated lands [157]. The challenge lies in the fact that plants can utilize only a small fraction of the applied phosphate fertilizers, and a significant portion of the soluble phosphorus becomes immobilized in the form of insoluble precipitates after reacting with Al^3+^ and Fe^3+^ ions in acidic soils [159].

The use of phosphate fertilizers over the last century has led to a significant increase in agricultural yields and production, but the future of these fertilizers is uncertain as phosphate mineral resources may be depleted soon [160]. Fortunately, soils host microorganisms, especially fungal and bacterial species, which act in association with plants (root systems, rhizosphere) to dissolve complex forms of phosphorus and other minerals necessary for plant growth [161].

**Figure 3.**
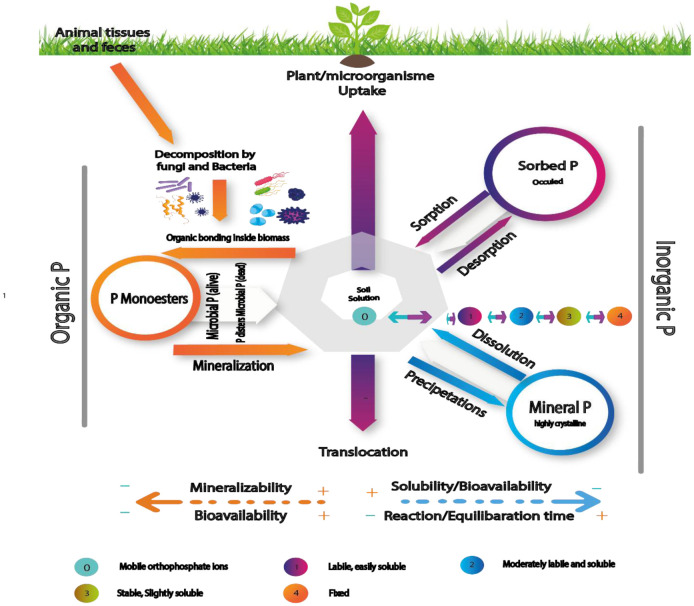
The phosphorus cycle in soil, showing main mobilization processes in soil and sources and reservoirs.

In the phosphorus cycle, organic phosphorus (P) can be released either as a byproduct of soil organic matter mineralization or through the action of specific enzymes regulated according to the demand for this nutrient. The key mechanism underlying inorganic phosphate solubilization entails the synthesis and exudation of organic acids by soil microorganisms (Figure 3). Typically, these organic acids originate from glucose oxidation [162]. To solubilize insoluble P, phosphate-solubilizing bacteria generate low molecular weight organic acids like acetate, gluconate, lactate, citrate, succinate, formic acid, and oxalic acid. These organic acids are responsible for the solubilization of insoluble phosphate [163]. Acidification of the medium by organic acids not only enables phosphate solubilization but also enables these organic acids to chelate iron and aluminum associated with phosphate [164]. Studies conducted by Mahdi et al. (2011) have revealed that these phosphate-solubilizing bacteria can liberate inorganic P from the mineral phosphate reservoir by substituting H^+^ for cations bound to phosphate. Moreover, certain soil bacteria utilize enzymes like phosphatases and phytases to mineralize organic phosphorus [165].

###### 3.3.2.3.2 Potassium solubilization

Potassium (K) is the third essential macronutrient required for plant growth, metabolism, and development. It plays a critical role in enhancing plant resistance to diseases, pests, and abiotic stress, and is involved in the activation of over 80 enzymes linked to key physiological processes such as energy metabolism, starch synthesis, nitrate reduction, photosynthesis, and sugar degradation [166]. In soils, potassium exists in both available and unavailable forms, with a large proportion bound in insoluble minerals such as micas, illite, and orthoclase [167]. To compensate for its limited bioavailability, potassium-based fertilizers are commonly applied to sustain crop productivity. However, environmentally sustainable alternatives are needed to reduce reliance on costly, imported fertilizers. Certain soil bacteria can enhance potassium availability by producing organic acids that promote the dissolution of insoluble potassium minerals, thereby making this nutrient more accessible to plants [168].

###### 3.3.2.3.3 Zinc solubilization

Zinc is a micronutrient, an indispensable element for life that cannot be synthesized by living organisms, necessitating a balanced supply even in small quantities. Zinc assumes a vital role in plant physiology and metabolism, contributing to their robust growth. It participates in processes such as photosynthesis, chlorophyll synthesis, plant reproduction, and autophagy. Moreover, zinc is recognized for its contribution to grain yield, seed development, and the defense mechanisms of plants against pathogens and herbivores [169].

Microorganisms can use various mechanisms to enhance zinc availability in the rhizosphere, thereby promoting its absorption by plants, particularly through acidification. Changes in soil pH strongly influence the mobilization of micronutrients. For example, a one-unit decrease in pH can increase zinc availability by a factor of one hundred [170]. Zinc-solubilizing microorganisms lower the rhizosphere pH through the production of organic acids and proton (H^+^) exudation, which facilitates zinc absorption by plants [171]. Other mechanisms include the production of siderophores (Saravanan et al., 2011), proton extrusion, the redox system on the microbial cell membrane, and ligand chelation [172].

##### 3.3.2.4. Biological nitrogen fixation

Nitrogen is an essential component of plant cells, present in DNA, RNA, proteins, amino acids, and more. Eukaryotic plants rely on bacteria for biological nitrogen fixation as they cannot break the triple bond between two nitrogen atoms. Diazotrophic bacteria capture and fix atmospheric nitrogen in soils, converting it into ammonia, which serves as the initial substrate for the nitrification process [173]. Nitrification, which entails the transformation of ammonium into nitrate, is executed by nitrifying bacteria such as *Nitrosomonas* spp. or *Nitrobacter* spp. [174].

Certain plant growth-promoting bacteria, including *Klebsiella* sp., *Acinetobacter* sp., *Bacillus pumilus* [175], *Azotobacter* spp. [63], *Burkholderia* [176], and *Pseudomonas* [177], have the ability to fix atmospheric nitrogen. PGPR with this capacity can enhance nitrogen uptake throughout the plant [175]. In addition to nitrogen fixation, PGPR participate in other reactions to complete the nitrogen cycle on Earth. Nitrogen-fixing rhizobia bacteria can form a highly regulated and specific symbiosis with legume roots [178]. Assessment of atmospheric nitrogen fixation by microorganisms can also be conducted through the identification of the *nifH* gene, which encodes the Fe protein of the nitrogenase enzymatic complex [179].

#### 3.3.3. Indirect mechanisms of PGPR action

In addition to directly promoting plant growth, PGPR can also indirectly enhance plant development by protecting against pests and environmental stressors. This protective function, known as biocontrol, involves the use of living microorganisms to suppress or eliminate phytopathogens, thereby reducing disease incidence and crop losses. Biocontrol is typically based on antagonistic interactions between organisms that share the same ecological niche [180] and includes mechanisms such as competition for nutrients and root colonization sites, the production of antimicrobial compounds, and the induction of systemic resistance (ISR) in host plants [181]. Among PGPR, species of *Pseudomonas* and *Bacillus*, as well as certain diazotrophic bacteria, are among the most extensively studied for their biocontrol potential [164],[182].

##### 3.3.3.1. Induction of systemic resistance

PGPR have the ability to provide plants with effective defense against a wide range of fungal, bacterial, and viral diseases through a phenomenon called induced systemic resistance (ISR), which persists for long periods after induction [183]. ISR strengthens plant cell walls and modifies their physiological and metabolic responses, enabling an increase in the synthesis of defensive chemicals when attacked by pathogens [184]. Many strains of PGPR can induce systemic resistance against a wide range of phytopathogens [185]. For instance, the induction of systemic resistance against the bean yellow mosaic potyvirus (BYMV) in faba bean (*Vicia faba* L.) has been investigated through seed inoculation with strains of *Pseudomonas fluorescens* and *Rhizobium leguminosarum* [63],[186]. Furthermore, it has been documented that the depletion of iron in the rhizosphere, resulting from the production of siderophores, can trigger systemic resistance [187]. It is worth noting that ISR is distinct from induced systemic tolerance (TSI), which is triggered by bacteria producing ACC, antioxidants, cytokinins, or volatile organic compounds [188].

##### 3.3.3.2. Antibiosis

PGPR employ multiple antagonistic mechanisms to suppress root pathogens, primarily through the synthesis of diverse antimicrobial compounds. These include volatile and non-volatile antibiotics, siderophores, lytic enzymes, hydrogen cyanide (HCN), and a variety of secondary metabolites [189]. Notably, PGPR genera such as *Pseudomonas* and *Bacillus* produce a wide array of antibiotic compounds, including ammonia, butyrolactones, 2,4-diacetylphloroglucinol (DAPG), kanosamine, oligomycin A, oomycin A, phenazine-1-carboxylic acid, pyoluteorin, pyrrolnitrine, viscosinamide, xanthobaccin, and zwittermicin A, which play pivotal roles in pathogen inhibition [190].

The biocontrol potential of these metabolites has been validated through genetic and molecular studies; for example, overexpression of DAPG in *Pseudomonas fluorescens* mutants enhances resistance to bacterial wilt in tomato caused by *Ralstonia solanacearum* [191], with seed inoculation by DAPG-producing endophytes achieving up to 70% disease reduction in eggplant [130]. Another critical biocontrol trait is HCN production, which exerts dual roles depending on concentration: it acts as a signaling molecule at low levels and confers herbivore resistance at higher levels [192]. HCN not only serves as a direct pathogen suppressor but also exhibits metal chelation properties and contributes to plant growth promotion when produced by biofertilizer strains [193]. Moreover, the combined production of HCN and ammonia by certain PGPR strains may exert synergistic effects, enhancing plant growth and metabolic activity [194].

##### 3.3.3.3. Competition for iron

Strong competition for iron acquisition in the rhizosphere has been demonstrated, as well as the stability of high-affinity ferric siderophores (pyoverdine). These siderophores are molecules that sequester iron (III) from the rhizosphere, thereby limiting its availability and inhibiting the growth of other pathogens [25]. The production of siderophores has been tested for its antagonistic activity against phytopathogens, its effect on iron nutrition, and wheat plant growth [195]. Bacteria capable of producing siderophores include *Streptomyces* spp., *Erwinia herbicola, E. amylovora, E. carotovora, Sinorhizobium meliloti, Rhizobium leguminosarum, Agrobacterium tumefaciens, E. chrysanthemi, Pseudomonas* spp., and *Bradyrhizobium japonicum* [196]. Some *Pseudomonas* species have a strong iron chelation capacity [197]. They can recognize and use siderophores produced by other strains, whereas these others cannot use their own siderophores. This feature could favor the colonization and competition of these strains for substrates over other microorganisms present in the rhizosphere. However, while siderophore production is an important mechanism for PGPR activity, it is rarely essential for biocontrol [198].

##### 3.3.3.4. ACC deaminase production

Bacterial ACC deaminase plays a critical role in stimulating plant growth by mitigating the detrimental effects of ethylene induced by environmental stresses [199]. The functioning of ACC deaminase involves the breakdown of 1-aminocyclopropane carboxylic acid, the precursor of ethylene, into α-ketoglutarate and ammonium ions, which serve as carbon and nitrogen sources for the bacterium harboring the enzyme. ACC deaminase activity is prevalent in the plant microbiome, particularly in demanding environments [200]. This underscores the significance of this activity in plant-PGPR interaction and communication. The initial report on the isolation of the ACC deaminase enzyme was documented in the soil bacterium *Pseudomonas* sp. and the yeast *Hansenula saturnus* (*Cyberlindera*). Subsequently, this enzyme has been frequently discovered in soil-associated plant bacteria, fungi, and yeasts (Nascimento et al., 2014). Researchers have characterized ACC deaminase in various bacterial strains from diverse origins [201]. Research has shown that *Arthrobacter protophormiae*, a bacterium containing ACC deaminase, interacts with other beneficial microorganisms, notably enhancing rhizobial nodulation and mycorrhizal colonization, which in turn promotes salt tolerance in *Pisum sativum* plants [202]. Similar observations have been documented for various rhizobium strains [203]. The first documentation of the presence of ACC deaminase in rhizobia was published in 2003 [204]. Before this, the enzyme had been documented in only bacteria, yeasts, and free-living fungi. These discoveries open new research avenues, particularly in understanding the role of these bacteria in atmospheric nitrogen fixation during symbiosis with legumes. More recently, researchers have identified ACC deaminase not only in the *Rhizobium* genus but also in other genera within the *Rhizobiaceae* family (*Rhizobium, Sinorhizobium*, and *Agrobacterium*), *Phyllobacteriaceae* family (*Phyllobacterium* and *Mesorhizobium*), and in *Azospirillum* [203].

#### 3.3.4. Quantifiable impacts of PGPR on agrochemical reduction

Meta-analyses and large-scale field trials provide robust evidence that PGPR can significantly reduce chemical fertilizer and pesticide dependency while maintaining or enhancing crop yields. The capacity for input reduction is clearly demonstrated in various cropping systems. For instance, research in tomato has established a “75% threshold” for integrated nutrient management, where supplementing 75% of the recommended fertilizer rate with PGPR inoculants produced yields equivalent to those from full (100%) fertilizer rates, demonstrating a viable 25% reduction in chemical inputs [205]. Moving beyond mere maintenance, certain PGPR applications have been shown to actively boost productivity; in multi-season wheat trials, specific PGPR consortia not only enabled reduced fertilizer inputs but also increased yields by up to 64% compared to uninoculated controls [206]. The benefits of PGPR extend beyond agronomic performance to include critical environmental advantages. Notably, studies in pasture and crop systems utilizing a 50:50 mix of organic and inorganic fertilizers with PGPR inoculation demonstrated a remarkable 95% reduction in nitrogen leaching compared to conventional fertilization, all without compromising biomass production [207]. Furthermore, the efficacy of PGPR is often enhanced under environmental stress, a crucial attribute for climate resilience. A comprehensive meta-analysis confirmed this, revealing that while PGPR increased root and shoot mass by 35% and 28% respectively under normal conditions, these effects were significantly amplified under drought stress [208].

## 4. Multi-omics approaches in understanding plant-bacteria-fungal interactions

### 4.1. Genomics insights

Advancements in sequencing technologies and omics tools have substantially deepened our understanding of plant-microbe interactions, particularly in terms of biodiversity and gene expression regulation. The microbial communities in the rhizosphere and surrounding soil are vital in shaping plant resistance to abiotic stresses. Microbiome-based multi-omics studies have shown significant potential in advancing rhizospheric science, enabling the detailed characterization of plant-associated beneficial microorganisms and their functional roles [209].

An illustrative example involves the application of next-generation sequencing (NGS) on DNA extracted from soil and the rhizosphere. This approach has been instrumental in inferring microbial communities, providing innovative avenues for harnessing the potential of plant-associated microorganisms. The utilization of NGS offers novel insights into the intricacies of plant-microbe interactions, allowing for a more comprehensive understanding of the structure, dynamics, and functional roles of microbial communities in the rhizosphere [209]. Extensive studies on transcriptional changes in gene expression levels have been conducted to elucidate the mechanisms governing metabolic switches, developmental differentiation, and natural product biosynthesis [210].

Compared to amplicon sequencing, shotgun metagenomics provides distinct advantages by reducing amplification biases and offering insights into the metabolic potential and functional capabilities of microbial communities. This approach enables whole genome recovery and a more comprehensive analysis of microbiome constituents. However, it is important to recognize challenges related to computational demands, susceptibility to contamination in low biomass samples, the impact of host DNA contamination, and the complexity of data and analytical processes [211].

### 4.2. Transcriptomics and proteomics analyses

Meta-transcriptomics and meta-proteomics are essential tools for understanding functional dynamics within complex environments, such as specific soil samples. Meta-transcriptomics focuses on assessing gene expression, while meta-proteomics offers a comprehensive analysis of all proteins present in a biomass. RNA sequencing (RNA-seq), a key technique in meta-transcriptomics, facilitates ecological studies of microbial communities. However, interpreting RNA-seq data can be challenging, particularly when a well-annotated reference genome is unavailable for mapping reads. The increasing availability of annotated transcriptomes in curated databases and advancements in robust de novo RNA-seq assemblers are helping to mitigate these challenges [209]. In addition to genomics and transcriptomics, proteomics is a powerful approach for unraveling the connections between metabolic pathways and the production of natural products. Through the comparison of protein expression levels, proteomics sheds light on the regulation of various pathways, identifying key components in natural product biosynthesis that can be targeted for rational engineering [210]. However, metatranscriptomics studies face ongoing challenges, such as the difficulty in preparing unbiased, high-quality RNA samples, the potential for significant host RNA contamination, suboptimal data analysis methods, and the biological interpretation of uncharacterized transcripts. Moreover, the integration of metatranscriptomics with other omics data, such as metaproteomics, remains a complex issue [211].

### 4.3. Metabolomics contributions

Metabolomics, entailing the measurement of low molecular weight metabolites, enables comparative analyses of biological samples, facilitating the identification of secondary metabolites associated with orphan biosynthetic gene clusters (BGCs) under diverse culturing conditions [210]. This integrative approach examines all small molecules within an organism, which are generated from the information flow contained in the genome, transcriptome, and proteome. By employing advanced technologies like ultra-high-pressure liquid chromatography paired with high-resolution mass spectrometry or nuclear magnetic resonance spectroscopy, metabolomics delivers a detailed chemical profile of thousands of compounds. This significantly enhances our understanding of the metabolic landscape [209].

In microbiome studies, metabolomics targeting small molecules with molecular weights below 2000 Da provides insights into the physiological state of microbial ecosystems. Analyzing the metabolome derived from microbial samples establishes a crucial link between microbial and genetic compositions, connecting phenotypes through metabolites. Microbiome metabolomics applications include elucidating unknown gene functions, characterizing environmental microbial communities and their functions, conducting molecular epidemiology studies, discovering novel enzymes, and identifying potential metabolite biomarkers [211].

Addressing methodological challenges, targeted metabolomics concentrates on a known set of annotated compounds, enabling precise quantitative analysis through comparisons with established libraries of analytical standards. Although less comprehensive than untargeted approaches, targeted metabolomics provides detailed information on numerous compounds, particularly suitable for examining known processes and responses. Conversely, untargeted metabolomics, with its unconstrained profiling of a myriad of compounds, facilitates broad comparisons within and between experimental units, leading to the discovery of pathways and compounds influencing biological processes. This untargeted approach proves indispensable for multi-omics studies, facilitating connections across genomic, metagenomic, volatilomic, and spectromic approaches [212].

### 4.4. Integration of multi-omics data

The integration of multiple omics analyses has advanced the study of interaction networks within agroecosystems. Ichihashi et al. (2020) performed a comprehensive multi-omics study in an arable field, incorporating various soil management practices. Their approach combined plant phenotype data with four distinct omics datasets: plant metabolome, rhizosphere microbiome, soil metabolome, and soil ionome. The findings revealed that soil management practices influenced several omics layers. By reconstructing the module structure of the agroecosystem from these multi-omics data, they discovered a novel interaction where soil choline was found to enhance crop growth. [28].

Advances in high-throughput sequencing technologies have revealed the astounding diversity and ubiquity of the microbial world in environmental samples. The multi-omics revolution has reshaped microbial ecology, highlighting that most microorganisms in ecosystems outnumber those accessible through cultivation by orders of magnitude. The “great plate count anomaly” has prompted innovative cultivation techniques, including microfluidics, cultivation chips, single-cell manipulation, and high-throughput cultivation [213].

A range of integrated multi-omics analysis tools that accommodate feature abundance have been developed to support combinations of omics data. These tools, including MOFA (Multi-Omics Factor Analysis), mixOmics, MiBiOmics, mmvec (Microbial Multimodal Variational Autoencoder), and mCIA (Multiple Co-Inertia Analysis), enable researchers to perform initial data analysis steps separately for each omics type.

MOFA is a framework that identifies hidden factors driving variability across omics layers, uncovering shared and unique patterns. mixOmics is an R package offering methods like canonical correlation analysis and sparse partial least squares to explore relationships between omics data types. MiBiOmics provides an intuitive platform for integrating microbiome data with other omics, enabling the analysis of interactions between microbial features and biological variables. mmvec uses machine learning to model associations and predict metabolite abundances from microbial features, while mCIA applies co-inertia analysis to find covariance between different omics datasets, aiding in the visualization of shared trends.

This approach helps in identifying the most variable feature groups across omics layers and exploring their relationships with specific phenotypes. For example, in studies of metabolites affecting flavor development and their association with particular microbial community members, researchers can combine amplicon sequencing with metabolomics and use mmvec to identify key co-occurring microbes and metabolites, revealing insights into microbe-metabolite interactions. Longitudinal studies can benefit from tools like PALM, which provides a robust means to examine temporal changes comprehensively. Additionally, COMBI facilitates the joint visualization of different omics features, aiding in the determination of associations between these features and phenotypes. Through these multi-omics analyses, significant advancements can be made in enhancing cheese quality, understanding flavor development factors, and guiding the selection of starter cultures or fermentation conditions [211].

### 4.5. Computational models for multi-omics integration

Computational paradigms for predicting microbial functions and plant phenotypes have facilitated a transition from simple correlation-based integration to mechanistic and predictive frameworks, enabling the rigorous interrogation of genomics, transcriptomics, proteomics, and metabolomics data [214]. These advanced models are critical for deciphering the complex, nonlinear, and hierarchical relationships inherent in multi-omics datasets, thereby providing superior accuracy in phenotype prediction and functional inference compared to traditional linear methods [215].

Deep learning architectures have emerged as particularly potent tools in this domain. For instance, the Deep Neural Network for Genomic Prediction (DNNGP) has been shown to dynamically learn high-level features from raw multi-omics matrices, significantly outperforming conventional models in predicting complex plant traits across diverse datasets [216]. Expanding on the utility of these architectures, Lhayani et al. (2025) demonstrated that deep learning models integrating morphological and molecular data specifically ITS2 and matK+rbcL barcodes can effectively bridge the gap between traditional and molecular taxonomy in the Fabaceae family, offering a novel approach to evaluate barcode discrimination efficiency [217]. Similarly, the application of nonlinear two-step frameworks and kernel machine methods enables the explicit modeling of interactions between distinct omics layers, such as the microbiome and metabolome, enhancing the resolution of plant phenotype predictions [218].

Complementing these data-driven approaches, mechanistic frameworks such as genome-scale metabolic models (GEMs) and constraint-based reconstruction provide a systems-level understanding of metabolic fluxes and protein activity [219]. The integration of transcriptomic and metatranscriptomic data into GEMs refines the prediction of microbial growth rates, metabolite exchanges, and community functions, particularly under fluctuating environmental conditions [220]. Furthermore, network-based integrative approaches enable the causal mapping of regulatory elements and molecular drivers, facilitating the identification of key biomarkers for agronomic traits and microbial metabolic interactions [221],[222]. Collectively, these computational advances are transforming systems biology by moving beyond association to causation, offering actionable insights for crop improvement and candidate gene discovery [223].

### 4.6. Future of multi-omics research

Climate change intensifies the impact of abiotic stresses on plants, limiting agricultural productivity [209]. Plants respond to these stresses through a polygenic network involving the detection of stress signals, signal transduction pathways, and the expression of stress-responsive genes, which collectively contribute to stress tolerance. The belowground microbiome is increasingly recognized as a valuable tool for enhancing stress tolerance, with mycorrhizal fungi and Actinomycetes identified as key plant allies. Employing a multi-omics approach can provide deeper insights into these beneficial interactions and facilitate their exploitation to improve plant resilience against abiotic stresses. Diverse omics approaches enable a comprehensive understanding of genetic backgrounds, gene expression changes, and microbial mediation of host plant responses to stress conditions.

Omics technologies have been pivotal in uncovering the key genes and pathways related to plant invasiveness. Genomic studies on invasive plants have elucidated the mechanisms driving their invasive traits [29]. By comparing invasive species with non-invasive counterparts, researchers have identified specific genes and pathways linked to invasiveness. These omics approaches also illuminate the intricate relationships between soil, microbiomes, and soil health, which are vital for maintaining terrestrial ecosystems and ensuring food security. Integrating omics techniques with ecological research is crucial for comprehensively understanding the complex interactions between invasive species and their environments. In the future, researchers should focus on advancing technologies for the analysis of complex omics data to further enhance our understanding of invasive species dynamics. To this end, shifting toward shotgun metagenomic sequencing will be pivotal. Unlike traditional amplicon-based methods, shotgun sequencing enables the comprehensive identification of soil microbial communities by capturing abundant and rare taxa, providing the high-resolution taxonomic profiles necessary to characterize different agricultural zones [224].

The challenge now is to scrutinize multi-omics datasets to address ecological and exploratory questions about microbiome members and their roles in natural habitats. Beyond taxonomy, functional annotation of metagenomic data enables the quantification of genes involved in critical soil functions, such as *nifH* (nitrogen fixation), *pqq* (phosphate solubilization), and *acdS* (plant growth promotion). Understanding the abundance and diversity of these functional markers is essential, as they directly correlate with soil fertility and crop yield [225],[226].

Multi-omics methods offer more than explanatory tools; they generate hypotheses awaiting testing through advanced cultivation technologies. Comparative analyses across these datasets can reveal a “core productive microbiome” a conserved set of microbial taxa and functional genes associated with high-yield environments. Identifying this core signature serves as a blueprint for rational agricultural zoning and the design of targeted bioinoculants [227].

From targeted isolation to high-throughput screening of colonies, multi-omics integration enhances success rates and reduces the search space in the quest for new microbial isolates [213].

While our understanding of plant-microbiome interactions has begun to unfold, how these interactions respond to climate change remains poorly understood [228]. Researchers should examine these interactions over time and space under multiple climate change scenarios, adopting a system-based, reductionist approach. Detailed insights into plant-microbiome interactions, coupled with predictive computational tools, are essential to anticipate climate change impacts on the plant-associated microbiome and enhance climate resiliency in plant communities.

## 5. Prospects and final remarks

Several gaps persist in our understanding of the long-term implications of specific PGPR strains on crops and under diverse environmental conditions. Furthermore, the intricate molecular mechanisms governing the interactions between leguminous plants and microbiota, which are crucial for mitigating soil degradation and addressing the challenges posed by climate change, require further exploration. Practical challenges related to the large-scale implementation of PGPR-based agricultural interventions, including concerns regarding scalability, cost-effectiveness, and compatibility with farming practices, remain significant obstacles to widespread adoption. Despite these challenges, the promising potential of PGPR in enhancing plant resilience to environmental stresses, improving nutrient availability, and bolstering disease resistance emphasizes the critical need for comprehensive and sustainable soil management strategies.

The utilization of bioinputs, particularly specific strains of PGPR, exhibits significant promise in ameliorating the degradation of agricultural land, advocating for regenerative agriculture, surmounting challenges associated with climate change, diminishing reliance on chemical inputs, and mitigating greenhouse gas emissions. These bioinputs present a sustainable paradigm, nurturing soil health, and contributing to the establishment of robust agro-ecosystems. In navigating the intricate landscape of contemporary agriculture, the exploration and integration of bioinputs emerge as a propitious avenue for addressing environmental concerns, augmenting agricultural sustainability, and ensuring the enduring vitality of ecosystems. Consequently, an imperative exists for sustained interdisciplinary research endeavors aimed at elucidating the underlying molecular mechanisms, employing advanced omics technologies, and computational modeling. Such concerted efforts can lay the foundation for the formulation of nuanced and efficient sustainable agricultural practices that fully exploit the potential of PGPR. The intricate dynamics characterizing root microbiota communities in environments constrained by resources underscore the criticality of integrated approaches and collaborative initiatives spanning diverse disciplines. By systematically addressing these challenges and gaps, we can cultivate innovative solutions to advance global food security and environmental sustainability in response to escalating pressures within the domains of agriculture and ecology.

## 6. Conclusion and future perspectives

The study of plant-microorganism interactions, particularly within the rhizosphere, has evolved from a niche discipline to a cornerstone of modern agricultural science. The intricate web of symbiotic and associative relationships between plants, bacteria like PGPR, and fungi such as arbuscular mycorrhizae forms the foundation of soil health, nutrient cycling, and plant resilience. As we have detailed, these microorganisms employ a vast arsenal of direct mechanisms such as nitrogen fixation, phosphate solubilization, and phytohormone production and indirect mechanisms, including induced systemic resistance and antibiosis, to foster plant growth and protect against environmental stressors. The application of multi-omics technologies has revolutionized our understanding, enabling us to decipher these complex interactions at a molecular level and paving the way for microbiome-based solutions to the pressing challenges of food security and environmental sustainability.

### 6.1. Future perspectives in microbial-based agricultural products

The next generation of microbial agricultural products is poised to be more sophisticated, targeted, and effective. We can anticipate several key trends:

#### 6.1.1. Customized microbial consortia

The future lies in moving beyond single-strain inoculants towards rationally designed synthetic communities. Rather than relying on individual microbial species, contemporary research emphasizes the deliberate assembly of compatible microorganisms that function synergistically to provide multifaceted plant growth-promoting effects, enhanced nutrient cycling, and superior resilience to environmental stresses. The rational design of these consortia, informed by ecological principles and genome-scale metabolic modeling, enables the selection of strains with complementary functions and minimal antagonistic interactions [229]. For instance, one consortium member may specialize in nitrogen fixation, while another solubilizes phosphate, and a third provides pathogen protection through antibiosis or induced systemic resistance, thereby creating a more resilient and functionally diverse microbial team.

Empirical evidence demonstrates the superiority of this approach: simplified yet representative bacterial communities derived from maize roots have successfully recapitulated key functions of complex natural microbiomes, validating the principle that synthetic assemblages can maintain ecosystem services with reduced complexity [229]. Furthermore, rationally designed microbial consortia have proven highly effective in challenging applications such as contaminated soil remediation, where coordinated metabolic activities of multiple strains achieve pollutant degradation outcomes unattainable by single isolates [230]. This consortium-based strategy promises enhanced functional redundancy, ecological stability, and robust performance under variable field conditions, addressing a critical limitation of single-strain inoculants that often fail to establish or persist in complex soil environments.

#### 6.1.2. Synthetic biology applications

Advances in synthetic biology and genome editing tools, particularly CRISPR-Cas systems, are revolutionizing our capacity to engineer microbes with enhanced or entirely novel plant growth-promoting traits. These precision molecular techniques enable the stable integration of beneficial genetic circuits that can improve nitrogen fixation efficiency across broader environmental ranges, produce specific antimicrobial compounds on demand in response to pathogen detection, or synthesize and deliver signaling molecules that precisely modulate plant physiological responses to stress [231]. The engineering of microbes with augmented capabilities for producing plant growth promoters and biocontrol agents represents a fundamental paradigm shift, transforming beneficial microorganisms into programmable biological factories capable of dynamic responses to plant signals and environmental cues.

Synthetic biology approaches now extend beyond simple genetic modifications to encompass the engineering of entire plant-associated microbiomes through bottom-up strategies by isolating and engineering strains with enhanced traits and top-down approaches that facilitate *in situ* modification of native microbial communities via horizontal gene transfer [232]. Advanced synthetic regulatory circuits and biosensors enable these engineered microbes to activate beneficial functions only when specific molecular triggers are detected, thereby increasing functional efficiency and minimizing unintended ecological effects. Such designer microbes will serve as the foundation for next-generation biofertilizers and biopesticides, offering precision agriculture solutions that can be tailored to specific crops, soil types, and climatic conditions.

#### 6.1.3. Precision microbiome engineering

The integration of metagenomic analysis with computational modeling and machine learning algorithms is catalyzing the development of field-specific microbial inoculants, heralding the emergence of a “microbiome-as-a-service” model for precision agriculture. This transformative approach recognizes that soil microbial communities exhibit substantial spatial heterogeneity and that optimal inoculant performance requires compatibility with the native microbiome. Through comprehensive metagenomic profiling, researchers can characterize the composition, functional potential, and interaction networks of indigenous soil microbial communities, thereby identifying specific functional gaps or imbalances that constrain plant productivity [233].

Farmers adopting this model will be able to submit soil samples for detailed microbiome analysis and receive customized microbial formulations designed to complement their field's microbial community, correcting deficiencies in nutrient cycling capacity, pathogen suppression, or stress tolerance functions. This precision design approach is grounded in the concept of core microbiomes evolutionarily conserved functional guilds that underpin sustainable agroecosystem functioning across diverse pedoclimatic zones [233]. By identifying which core microbial functions are absent or underrepresented in a particular field's microbiome and selectively introducing appropriate microbial taxa, targeted interventions can restore critical ecosystem services without disrupting beneficial indigenous communities. Research priorities have been systematically established for harnessing plant microbiomes in sustainable agriculture, emphasizing the imperative need for predictive frameworks that mechanistically link microbial community structure to agronomic outcomes [234].

Advancements have integrated multi-omics and artificial intelligence to predict (Genotype × Environment) interactions with unprecedented precision [235]. Rizwan et al. highlight the use of machine learning algorithms to model how specific microbial genotypes adapt to variable agro-ecologies, emphasizing the molecular basis of environmental resilience. By utilizing big data to decipher these complex interactions, researchers can now develop “climate-smart” predictive frameworks. These models mechanistically link microbial community structure to agronomic outcomes, enabling the selection of inoculants that are robust against abiotic stresses like drought and heat, thereby ensuring consistent performance across environments [236].

#### 6.1.4. Advanced formulation technologies

To ensure the viability and field efficacy of these sophisticated microbial products, significant advances in formulation science are essential. Innovations in microencapsulation technologies wherein beneficial microbes are embedded within protective polymeric matrices such as alginate, polyvinyl alcohol, or chitosan-based biopolymers provide critical protection against ultraviolet radiation, desiccation, temperature extremes, and other environmental stressors encountered during storage, transport, and post-application establishment phases [237]. Microencapsulation not only extends shelf-life substantially but also enables controlled release of microbial cells in the rhizosphere, thereby improving colonization efficiency and functional persistence under field conditions.

Complementing microencapsulation, advanced seed coating technologies have emerged as particularly promising delivery systems for beneficial microorganisms in agricultural applications. Seed coating ensures uniform distribution of microbial inoculants in intimate proximity to emerging roots, facilitating early colonization and maximizing the window for beneficial plant-microbe interactions during the critical seedling establishment phase [238]. Modern seed coating formulations incorporate sophisticated polymeric matrices, adhesive binders, and nutritional carriers that enhance microbial survival on seed surfaces and support rapid rhizosphere colonization following germination. Empirical studies demonstrate that such bioformulations markedly improve germination rates, seedling vigor, and stress tolerance, particularly under challenging conditions such as salinity or drought stress [238].

Bioencapsulation strategies utilizing controlled-release formulations and biodegradable carriers have demonstrated marked improvements in microbial survival during extended storage periods and enhanced post-inoculation establishment in soil, thereby bridging the persistent gap between laboratory efficacy demonstrations and consistent field performance [237]. These innovations in formulation science are critical enablers for translating the biological potential of beneficial microorganisms into commercially viable, farmer-friendly products that can be integrated seamlessly into agricultural practices. Future developments will likely focus on intelligent delivery systems that respond to specific rhizosphere conditions, further optimizing the timing and localization of microbial release. Formulation science is undergoing a paradigm shift from bulk encapsulation to precision engineering at the single-cell level. Rheem et al., (2025) reviewed the emergence of Single-Cell Nanoencapsulation (SCNE), a process that creates “artificial spores” by wrapping individual cells in functional nanoshells, imparting exogenous traits such as extreme stress resistance without genetic modification [239]. Building on this, Han et al. (2025) demonstrated a specific application using metal-organic complexes (Fe^3+^BHC) to encase *Saccharomyces cerevisiae*. This approach not only ensures cytoprotection against heavy metals and antibacterials but also introduces a “smart” release mechanism where the protective shell degrades controllably in response to phosphate triggers in the rhizosphere [240]. These innovations represent a move toward programmable, intelligent delivery systems for agricultural microbes.

In conclusion, the soil-plant-microbe nexus represents a frontier of immense opportunity. The detailed mechanisms of PGPR and symbiotic fungi, now being unraveled with unprecedented clarity through multi-omics approaches, offer a blueprint for the next green revolution. While challenges in translating laboratory success to consistent field performance remain, the trajectory is clear. By harnessing the power of these beneficial microorganisms through rational design, synthetic biology, precision engineering, and advanced formulation technologies, we can develop agricultural systems that are not only highly productive but also self-sustaining and environmentally harmonious. Continued interdisciplinary research, bridging microbiology, plant science, ecology, synthetic biology, and data science is essential to fully realize this potential and cultivate a future where agriculture works in concert with nature, not in opposition to it.

## Use of AI tools declaration

The authors declare they have not used Artificial Intelligence (AI) tools in the creation of this article.


