# Supplementary material for: Understanding plant-microorganism interactions: The key roles of soil, rhizosphere, and direct and indirect mechanisms
Source: AIMS Microbiol. 2025 Dec 19;11(4):1035–78. doi: 10.3934/microbiol.2025046 (PMC12782948; doi:10.3934/microbiol.2025046)
Supplement: Supplementary file 1 [file microbiol-11-04-046-s001.pdf]

AIMS Microbiology

AIMS Microbiology, 11(4): 1035–1078.
DOI: 10.3934/microbiol.2025046
Received: 23 September 2025
Revised: 12 December 2025
Accepted: 16 December 2025
Published: 19 December 2025

https://www.aimspress.com/journal/microbiology

Review

Understanding plant-microorganism interactions: The key roles of soil, rhizosphere, and direct and indirect mechanisms

Mohamed Hnini[1,2,*], Karim Rabeh[3] and Malika Oubohssaine[1]

[1] Microbiology and Molecular Biology Team, Center of Plant and Microbial Biotechnology, Biodiversity and Environment, Faculty of Sciences, Mohammed V University of Rabat, Avenue Ibn Battouta, BP 1014, Rabat 10000, Morocco
[2] Research Team in Science and Technology, High School of Technology Laayoune, Ibn Zohr University, Morocco
[3] Oasis System Research Unit, Regional Center of Agricultural Research of Errachidia, National Institute of Agricultural Research, PO. Box 415, Rabat 10090, Morocco

* **Correspondence:** Email: hnini007@gmail.com, m.hnini@uiz.ac.ma.

Table S1. Direct and indirect mechanisms of Plant Growth-Promoting Rhizobacteria (PGPR).

Type of Mechanism	Specific Mechanism	Description and Benefits to Plants	Examples of Genera/Species	References
Direct	Phytohormone Production	PGPR synthesize plant hormones (e.g., indole-3-acetic acid, IAA) that regulate growth and stress responses, enhancing root elongation, nutrient uptake, and biomass production.	*Rhizobium*, *Bradyrhizobium*, *Azospirillum*, *Bacillus*, *Pseudomonas*	(Eichmann et al., 2021; Rolon-Cardenas et al., 2022; Khoshru et al., 2020;; Spaepen et al., 2014; Maheshwari et al., 2015)
	Iron Acquisition via Siderophores	Siderophores chelate Fe^{3+} in low-availability soils, improving iron uptake for both bacteria and plants, enhancing health and productivity.	*Streptomyces spp.*, *Erwinia spp.*, *Rhizobium leguminosarum*, *Agrobacterium tumefaciens*, *Pseudomonas spp.*, *Bradyrhizobium japonicum*	(Albelda-Berenguer et al., 2019; Guerinot, 1994;; Sadaghiani and Barin, 2008; Babalola et al., 2021)
	Mineral Solubilization	Soil microbes solubilize insoluble minerals, increasing nutrient availability to plants.	Soil bacteria and fungi	(Whitelaw, 1999)
	Phosphate Solubilization	PGPR release organic acids and enzymes (phosphatases, phytases) to convert insoluble phosphate compounds into bioavailable forms.	Soil bacteria and fungi	Karpagam and Nagalakshmi, 2014;; Khatoon et al., 2020;; Spaepen et al., 2009; Mahdi et al., 2011)
	Potassium Solubilization	Certain bacteria solubilize K-bearing minerals via organic acid production, enhancing plant K uptake and stress resistance.	Soil potassium-solubilizing bacteria	(Almeida et al., 2015; Sahu et al., 2021; Etesami et al., 2017)
	Zinc Solubilization	PGPR enhance Zn availability through acidification, siderophore production, redox reactions, and ligand chelation, promoting plant growth.	Zinc-solubilizing microorganisms	(Cabot et al., 2019; Havlin et al., 2016;; Saravanan et al., 2011; Dhaked et al., 2017)

Continued on next page

Table S1. Direct and indirect mechanisms of Plant Growth-Promoting Rhizobacteria (PGPR).

Type of Mechanism	Specific Mechanism	Description and Benefits to Plants	Examples of Genera/Species	References
Direct	Biological Nitrogen Fixation (BNF)	Diazotrophic PGPR convert atmospheric N_2 into NH_3, making nitrogen accessible to plants, particularly in legume symbiosis.	*Klebsiella sp.*, *Acinetobacter sp.*, *Bacillus pumilus*, *Azotobacter spp.*, *Burkholderia*, *Pseudomonas*, rhizobia (e.g., *Rhizobium*, *Sinorhizobium*)	(Heil et al., 2016; Kuan et al., 2016; Bhattacharyya and Jha, 2012;;; Gamalero and Glick, 2011; Nascimento et al., 2014)
**Indirect**	Biocontrol of Pathogens	PGPR suppress pathogens through competition, production of antimicrobials, and triggering plant defenses, thereby reducing disease incidence.	*Pseudomonas spp.*, *Bacillus spp.*, diazotrophic bacteria	(Ahmad and Aqil, 2007; Haas and Défago, 2005;; Spaepen et al., 2009)
	Induced Systemic Resistance (ISR)	ISR enhances plant defenses, reinforcing cell walls and metabolic pathways to resist a broad spectrum of pathogens.	*Pseudomonas fluorescens*, *Rhizobium leguminosarum*; also triggered by siderophore-mediated iron depletion	(Singh et al., 2021; Abdelkhalek et al., 2022;; Weyens et al., 2013)
	Antibiosis (Antimicrobial Compound Production)	PGPR produce antibiotics, lytic enzymes, siderophores, HCN, and secondary metabolites that inhibit root pathogens.	*Pseudomonas*, *Bacillus*; HCN-producing strains also reported	(Sehrawat et al., 2022;; Rijavec and Lapanje, 2016; Agbodjato et al., 2015)
	Competition for Iron	High-affinity siderophores sequester iron, limiting pathogen access and thereby inhibiting their growth.	*Streptomyces spp.*, *Erwinia spp.*, *Sinorhizobium meliloti*, *Pseudomonas spp.*, *Bradyrhizobium japonicum*	(Kloepper et al., 1980; Maheshwari, 2010)
	ACC Deaminase Production	ACC deaminase degrades ACC (ethylene precursor), reducing ethylene levels under stress and promoting plant growth. Enhances nodulation and mycorrhizal colonization.	*Pseudomonas sp.*, *Hansenula saturnus*, *Arthrobacter protophormiae*, *Rhizobium*, *Sinorhizobium*, *Agrobacterium*, *Phyllobacterium*, *Mesorhizobium*, *Azospirillum*	(Ekimova et al., 2018.Barnawal et al., 2014).

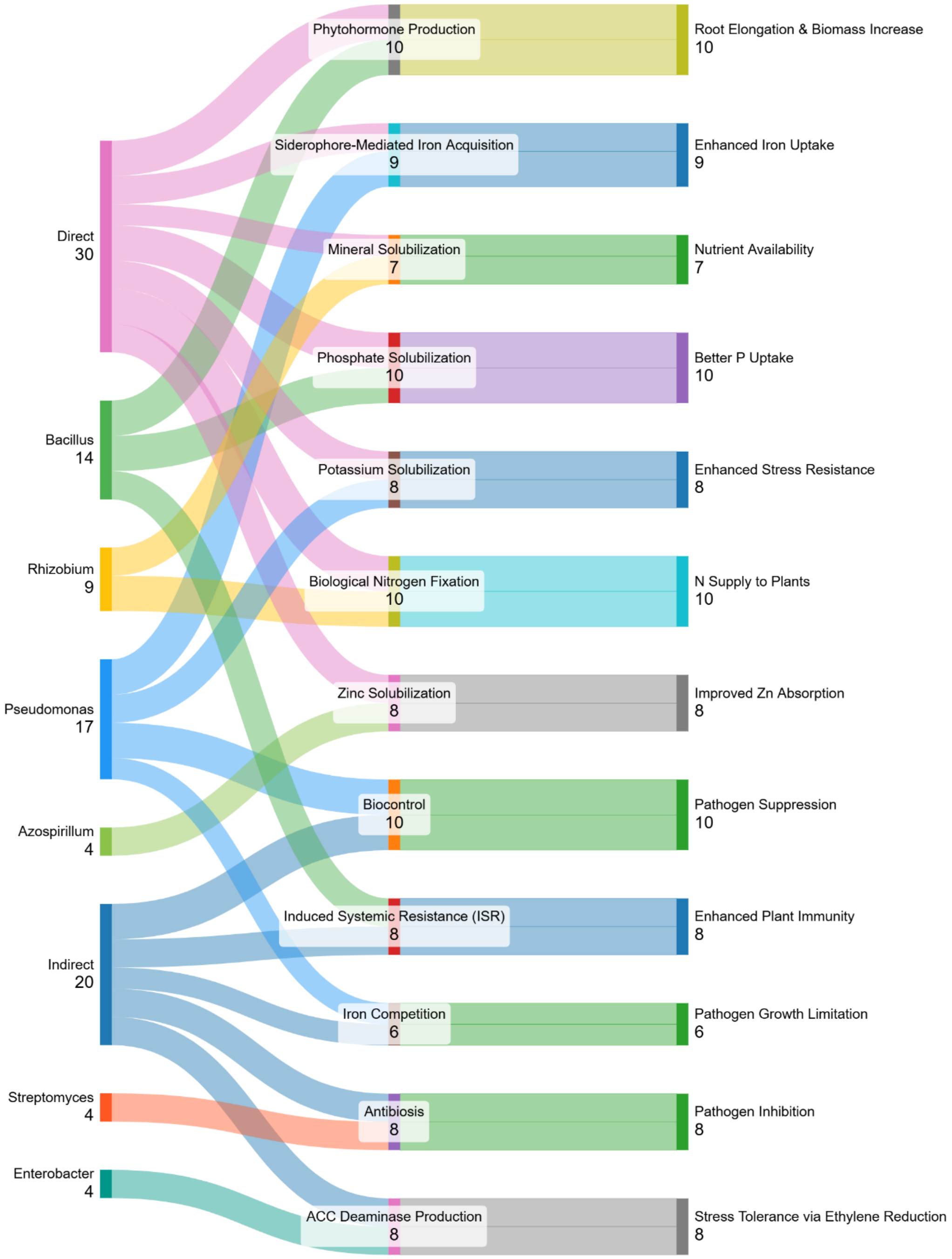

Figure S1. Sankey diagram illustrating direct and indirect mechanisms of plant growth-promoting rhizobacteria (pgpr) in enhancing plant growth and resilience.

AIMS Press

© 2025 the Author(s), licensee AIMS Press. This is an open access article distributed under the terms of the Creative Commons Attribution License (https://creativecommons.org/licenses/by/4.0)
